# The complete mitochondrial genome of *Eremias dzungarica* (Reptilia, Squamata, Lacertidae) from the Junggar Basin in Northwest China

**DOI:** 10.1080/23802359.2021.1923417

**Published:** 2021-06-15

**Authors:** Song Wang, Jinlong Liu, Bin Zhang, Xianguang Guo

**Affiliations:** a College of Life Sciences and Technology, Inner Mongolia Normal University, Hohhot, China; b Chengdu Institute of Biology, Chinese Academy of Sciences, Chengdu, China

**Keywords:** Mitochondrial genome, next-generation sequencing, phylogenetic tree, *Eremias dzungarica*, viviparity

## Abstract

The complete mitochondrial genome (mitogenome) of *Eremias dzungarica* from the Junggar Basin in Northwest China was determined for the first time by next-generation sequencing. The total length is 19,899 bp, containing13 protein-coding genes (PCGs), two ribosomal RNA genes, 22 transfer RNA genes and one control region. This gene arrangement is similar to the typical mtDNA of vertebrates. The 13 concatenated PCGs were used to perform Bayesian phylogenetic analyses together with several congeners as well as ten representative lacertids with mitogenome data available in GenBank. The resulting phylogenetic tree supports the monophyly of viviparous species in *Eremias*, with *E. dzungarica* being more closely related to *E. przewalskii* than to *E. multiocellata.* The mitogenome of *E. dzungarica* will provide fundamental data for the exploration of the mitogenome evolution in lacertids.

The Dzungarica Racerunner, *Eremias dzungarica*, is a newly described species among the viviparous group in genus *Eremias* (Orlova et al. 2017). This species is characterized with habitat preference to rocky hill and gravel ravines in western Mongolia at high elevations (2400–2600 m above sea level), but it can also penetrate into low-altitude sandy dune areas in eastern Kazakhstan (400–1000 m above sea level). Its potential occurrence in the vast territories of northern Junggar Basin in Northwest China was confirmed by our team through extensive field expedition in combination with DNA barcoding (Liu et al. 2021). So far, there is still limited understanding about the mitochondrial genome (mitogenome) evolution in *Eremias* lizards, even in lacertid lizards (Reptilia, Squamata, Lacertidae).

In this study, we produced for the first time the complete sequence of the mitogenome of *E. dzungarica* by next generation sequencing through the Illumina NovaSeq platform. The specimen (voucher number GXG667) was collected from Hoboksar Mongol Autonomous County (N46.76°, E85.75°) located in the Junggar Basin in July 2019, which is the second largest inland basin in Northwest China. Its liver tissue was fixed with 95% ethanol, and stored at −20 °C in the herpetological collection, Chengdu Institute of Biology, Chinese Academy of Sciences. A small amount of liver tissue was shipped to Genepioneer Biotechnologies (Nanjing, China) for genomic extraction and 150-base-pair paired-end library construction as well as sequencing.

The raw data was processed with fastp v.0.20.0 (https://github.com/OpenGene/fastp; Chen et al. 2018) by trimming adapters and primers, filtering reads with phred quality < Q5, and filtering reads with N base number > 5. *De novo* assembly of clean data (17,198,388 pair-end reads) was performed using SPAdes v.3.10.1 (http://cab.spbu.ru/software/spades/; Bankevich et al. 2012) in order to get the whole mitogenome. The average coverage of reads aligned on the mitochondrial genome was 1053.1545. Then, we took a similar strategy to that in Chen et al. (2019) to blast the mitogenome against the published reference sequences of *Eremias multiocellata* (accession number KM257724; Tong et al. 2016) for comparison, alignment and annotation. Subsequently, the mitogenome was also annotated with MITOS Web Server (http://mitos.bioinf.uni-leipzig.de/index.py; Bernt et al. 2013). Specifically, 22 tRNA genes were identified by using the web server of tRNA scan-SE (http://lowelab.ucsc.edu/tRNAscan-SE/; Lowe and Chan 2016). The base composition was calculated in MEGA v.7.0 (Kumar et al. 2016).

The entire mitogenome of *E. dzungarica* was 19,899 bp in length, which was composed of 28.4％ T, 26.8％ C, 31.6％ A, 13.2％ G. All of 37 genes were completely recovered including two ribosomal RNA genes, 13 protein-coding genes (PCGs), 22 transfer RNA (tRNAs), and a control region (CR or D-loop). The gene content, arrangement, and composition exhibited a typical vertebrate mtDNA features. The majority of the genes in the mtDNA of *E. dzungarica* were distributed on H-strand, except for *ND6* gene and eight tRNAs (*tRNA-Glu, Ala, Asn, Cys, Tyr, Ser^[UGA]^, Gln,* and *Pro*). In the 13 PCGs, the shortest was *ATP8* gene (162 bp) and the longest was *ND5* (1824 bp). Eleven PCGs used ATG as start codons, the remaining two (*COX1, ND1*) used GTG as start codons. As for stop codon, five PCGs (*ND5, ND4L, ND1, ATP6, ATP8*) used TAA as stop codons; five PCGs (*ND2, ND3, ND4, COX2, COX3*) used T as an incomplete stop codon; two PCGs (COX1, ND6) used AGG as stop codons; the last one (*Cytb*) used TAG as stop codon. In addition, 12S rRNA, 16S rRNA, and D-loop were 951 bp, 1559 bp, and 4496 bp in length, respectively.

The 13 concatenated PCGs of *Eremias* spp. and other representative lacertids available in GenBank were used to reconstruct phylogenetic tree for assessing the mitochondrial sequence authenticity of *E. dzungarica* and its phylogenetic position. The plug-in programs in PhyloSuite v.1.2.1 (Zhang et al. 2020) were used for gene partitioning, model selection, and tree reconstruction. The best-fitting substitution models and partitioning schemes were selected in PartitionFinder v.2.1.1 (Lanfear et al. 2017) using the Bayesian information criterion. Partitioned Bayesian analyses were conducted using MrBayes v.3.2.6 (Ronquist et al. 2012). Two independent runs were carried out with four Monte Carlo Markov chains (MCMCs) for ten million generations with parameters and topologies sampled every 1000 generations. Convergence of the runs was assessed by the standard deviation of split frequencies (<0.01). A 50% majority-rule consensus tree and posterior probability (PP) of clades were assessed by combining the sampled trees from the two independent runs after a 25% burn-in phase.

As shown in Figure 1, the resulting phylogenetic tree recovered monophyly of genus *Eremias* and confirmed that viviparous species form a monophyletic group (Guo et al. 2011; Orlova et al. 2017) with strong support. *Eremias dzungarica* was more closely related to *E. przewalskii* than to *E. multiocellata.* The mitogenome of *E. dzungarica* will provide fundamental data for exploration of the mitogenome evolution in lacertids (Lacertidae) in general, and of the viviparity evolution in racerunner lizards (*Eremias*) in particular.

**Figure 1.**
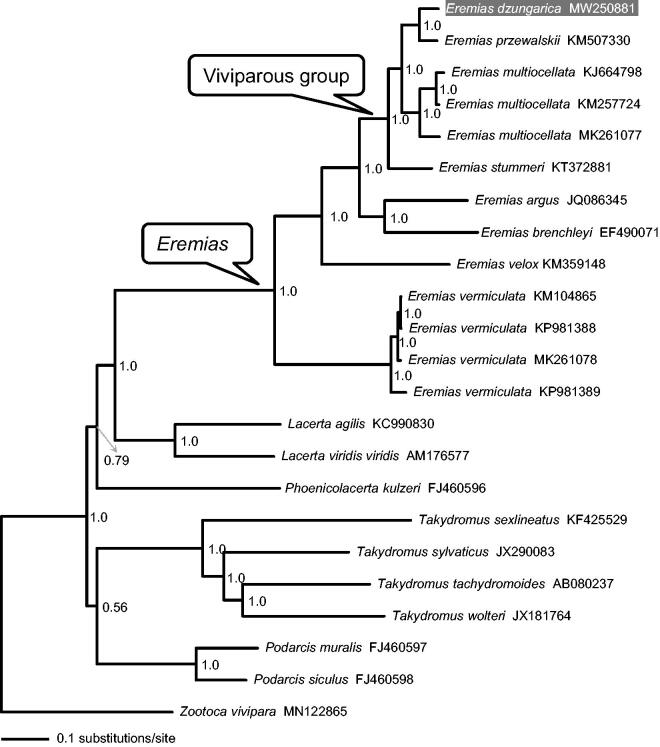
A majority-rule consensus tree inferred from Bayesian inference using MrBayes with the best models for each partition, based on the PCGs of *Eremias* spp. and ten other representative lacertids retrieved from GenBank. The phylogenetic placement of *E. dzungarica* is highlighted. GenBank accession numbers are given with species names. Node numbers show Bayesian posterior probabilities. Branch lengths represent means of the posterior distribution.

## Data Availability

The genome sequence data that support the findings of this study are openly available in GenBank of NCBI at (https://www.ncbi.nlm.nih.gov/nuccore/ MW250881) under the accession no. MW250881. The associated BioProject, SRA, and Bio-Sample numbers are PRJNA714298, SRR13960197, and SAMN18299467, respectively.
